# IMPACT OF OACS ON OUTCOMES OF COVID-19 INFECTION IN OLDER ADULTS WITH HISTORY OF ATRIAL FIBRILLATION

**DOI:** 10.1093/geroni/igac059.3045

**Published:** 2022-12-20

**Authors:** Ricardo Criado Carrero, Andrew Carl Crawford, Ali Vaeli Zadeh, Alan Wong, Ana De, Diego Diaz, Elias Collado, Joshua Larned

**Affiliations:** University of Miami/Holy Cross Hospital, Fort Lauderdale, Florida, United States; University of Miami/Holy Cross Hospital, Fort Lauderdale, Florida, United States; Holy Cross Health-Jim Moran Cardiovascular Research Institute, Fort Lauderdale, Florida, United States; Holy Cross Health-University of Miami, Fort Lauderdale, Florida, United States; University of Miami/Holy Cross Hospital, Fort Lauderdale, Florida, United States; University of Miami/Holy Cross Hospital, Fort Luderdale, Florida, United States; Holy Cross Health-Jim Moran Cardiovascular Research Institute, Fort Lauderdale, Florida, United States; Holy Cross Health- University of Miami, Miami, Florida, United States

## Abstract

**Background:**

Age, atrial fibrillation (AF), and COVID-19 infection predispose patients to hypercoagulability and poor outcomes. It is unclear if older adults with AF and COVID-19 infection would benefit from oral anticoagulants (OACs).

**Methods:**

A retrospective study was conducted using the PearlDiver database (PearlDiver Technologies, Fort Wayne, IN). Using ICD-10 codes, adults aged 65–75 and Elixhauser Comorbidity index(ECI) >4 with a history of AF admitted for COVID-19 were identified. The use of OACs for 6 months before the index event was used to split the cohort into two propensity score-matched groups considering age, gender, and ECI. Records from both groups were reviewed for multiple outcomes during the same admission. Pearson’s chi-squared test was used to compare groups. The strength of association was reported using Risk Ratios (RR). A p-value < 0.05 was deemed significant.

**Results:**

We compared 16,967 individuals in both anticoagulated and non-anticoagulated groups. Anticoagulated patients had a lower risk of mortality (RR=0.11, p=0.026), and a higher risk of 30-day all-cause readmission(RR=1.12, p < 0.0001). However, there were no differences in ICU admission, gastrointestinal bleeding, intracranial hemorrhage, thromboembolic events, or length of hospitalization.

**Conclusion:**

Compared to non-anticoagulated patients, older adults with a history AF on chronic oral anticoagulants had a lower risk of all-cause mortality, and higher risk of 30-day all-cause readmission. This information would help clinicians decide whether to prescribe OACs to this population of patients.

